# Advances in Multifunctional Polymer-Based Nanocomposites, 2nd Edition

**DOI:** 10.3390/polym18202473

**Published:** 2026-10-10

**Authors:** Jia-Wun Li, Chih-Wei Chiu

**Affiliations:** Department of Materials Science and Engineering, National Taiwan University of Science and Technology, Taipei 10607, Taiwan

## 1. Introduction

“Advances in Multifunctional Polymer-Based Nanocomposites (2nd Edition)” highlights recent advances, emerging research directions, and significant findings in materials science and engineering. Multifunctional polymer nanocomposites combine the processability and mechanical compliance of polymers with the functions introduced by nanoscale components. Designing these nanocomposites mandates control of the filler geometry, surface chemistry, dispersion, and processing history. Reinforcement, charge transport, thermal conduction, and optical response depend on the interactions of these components with the matrix and among one another. Research on dielectric interfaces, continuous ceramic frameworks, and ordered hydrogels illustrates the importance of interfacial bonding and network organization [1,2,3], while exposing a recurring design challenge: improving one property can alter another, making the balance among function, durability, and manufacturability central to material development. Transport properties show why spatial organization matters. Aligned boron nitride nanosheets and graphene networks allow heat flow control in polymer composites [4,5]. Related systems address solar absorption and infrared emission or thermal management and electromagnetic shielding [6,7]. Heterostructured ZnO–ZnS fillers have also been investigated for improving the dielectric breakdown strength and electrostatic energy density [8]. These examples connect local interfacial interactions with macroscopic performance. For optoelectronic and conductive composites, the relevant question is therefore how filler concentration and arrangement influence the response, including their competing effects on optical transparency, flexibility, or processing.

Broadly, the studies presented here highlight a general design principle for multifunctional polymer nanocomposites: macroscopic performance is governed not only by the intrinsic properties of the constituents but also by interfacial interactions, spatial organization, and the formation of effective transport networks. As research has expanded beyond single-property enhancement toward the integration of multiple functions, these design concepts have been extended to energy-storage and separation systems. Thus, polymer-based hybrid systems can reveal how interfacial chemistry and nanoscale organization can regulate transport processes. Composite polymer electrolytes and two-dimensional fillers illustrate strategies for facilitating ion transport [9,10], while nanoclay-reinforced systems connect interfacial chemistry with electrochemical function [11]. Despite differing in composition and application, these materials collectively emphasize the importance of continuous and accessible transport pathways in determining functional performance. Similar considerations arise in membrane systems, where two-dimensional polymer nanosheets provide additional structural building blocks [12], while aqueous-phase fabrication addresses processing and service conditions [13]. PVDF-based composites investigated for wastewater treatment and energy storage further expand this application space [14]. Beyond energy-storage and separation applications, flexible sensing represents another important area where functional response must be balanced with mechanical compliance. Piezoelectric polymer nanocomposite membranes, conductive organohydrogels, reinforced cellulose membranes, and antibacterial conductive composites have been studied for pressure, strain, or temperature sensing [15,16,17,18]. Hierarchical conductive networks can support touch sensing [19], while integrated nanocomposite sensors enable monitoring of respiratory activities [20]. Combining sensing systems with machine learning has expanded their application to respiratory analysis and human–machine interfaces [21,22]. CNT-network actuators offer a related route to mechanical output [23]. Across these applications, sensitivity must be considered alongside hysteresis, environmental stability, calibration, and repeatability under deformation. Manufacturing strategies also determine whether these functions can be translated into reproducible and practically useful components. Additive manufacturing generates complex geometries in functional polymers and nanocomposites [24]. Covalent functionalization of graphene oxide facilitates its incorporation into stereolithographically printed structures [25]. Bio-based photopolymers containing dynamic imine bonds provide opportunities for reprocessing [26], while green-solvent-processed cellulose/graphene oxide films combine reinforcement with thermal and ultraviolet resistance [27]. These developments motivate the consideration of renewable feedstocks, dispersion chemistry, processing energy, and end-of-life options alongside performance. Renewable origin alone does not establish environmental superiority; the processing route and service life also require assessment. Alongside advances in materials processing and sustainability, data-driven approaches are gaining relevance in the design and evaluation of multifunctional polymer systems. Machine learning helps correlate polymer composition and processing with the measured properties [28]. For sensing applications, this convergence of functional materials and data analysis is illustrated by recent developments in surface-enhanced Raman scattering (SERS). Plasmonic gold nanopockets have been combined with computational classification for microplastic detection [29]. Flexible SERS-active polymer nanofibers extend this approach to wearable sweat analysis [30], while biomedical applications require reproducible substrates and validation in relevant sample matrices [31]. Lu et al. reported a dynamic SERS platform integrating multiscale nanostructural engineering, programmable surface energy, and artificial intelligence-assisted discrimination of *Helicobacter pylori* [32]. This work links sensing interface control with spectral interpretation and provides a relevant perspective on the integration of material function and data analysis.

The first edition of this Special Issue, “Advances in Multifunctional Polymer-Based Nanocomposites”, collated 15 research and review contributions covering reinforcement, thermal and optical properties, sensing, energy conversion, and other related applications [33]. Building on this foundation, the second edition presents seven contributions addressing PVDF-based optical materials, bio-polyamide hybrids, aerogel and xerogel electrodes, toughened thermoplastic blends, asymmetric membranes, antibacterial ZnO/polymer systems, and printable CNT composites. These studies examine different functions while linking material composition/architecture to performance. The following overview summarizes their principal findings and identifies implications for the development of reproducible, application-oriented polymer materials.

## 2. Overview of Published Articles

Taha et al. investigated the optical properties of solution-cast PVDF/SrTiO_3_/CNT films at nanofiller contents of 0, 0.1, 0.3, and 0.7 wt% [34]. Structural and morphological analyses demonstrated changes in the polymer crystalline organization and spherulitic morphology. The tabulated optical parameters indicated that at 0.1 wt%, the indirect and direct band gaps increased from 5.27 and 5.56 eV to 5.50 and 5.70 eV, respectively. However, the estimated static refractive index decreased from 2.42 to 2.32 at 0.1 wt%, increasing to 3.06 and 4.27 at 0.3 and 0.7 wt%, respectively. These results demonstrate composition-dependent optical tuning and motivate further evaluation for optoelectronic applications. Device performance is not established by film characterization alone.

Teodorescu et al. addressed sustainable reinforcement through a feather-keratin/halloysite nanohybrid incorporated into a bio-based polyamide [35]. Keratin was combined with untreated or surface-treated halloysite to form two nanohybrids, which were melt-processed with the polyamide by extrusion. Both nanocomposites contained 5 wt% keratin and 1 wt% halloysite. The formulation based on treated halloysite, PA–KCHE, exhibited the most uniform nanohybrid dispersion and strongest component interactions. Compared to the unmodified matrix, its elastic modulus and surface hardness increased by approximately 75% and 30%, respectively, along with enhanced scratch resistance. By valorizing feather waste without excluding industrial melt-processing, this study provided a credible pathway from bio-derived constituents to mechanically robust nanocomposites for automotive applications.

Tran et al. reviewed green aerogel- and xerogel-based electrodes for supercapacitors [36]. The review covered synthesis and drying routes, porous structures, and the incorporation of polymers, metal oxides, etc., to modify the electrochemical behavior. Microwave heating was highlighted as a promising means of shortening processing time and improving heating uniformity, while pyrolysis temperature was identified as an important parameter governing gel morphology. The authors focused on flexible electrodes that retain capacitive performance under bending, hierarchical pores in biomass-derived materials, and the need for industrial-scale synthesis. The review thus connects precursor selection and processing with electrode architecture, while identifying biomass and organic waste as potential resources for further electrode development.

Markowski et al. explored MMT, HNTs, and two types of CNTs as phase-structure modifiers in impact-toughened polyethylene terephthalate glycol/polycarbonate blends [37]. Introducing the EBA-GMA impact modifier increased the Charpy impact strength of the reference PETG/PC blend from approximately 1.5 to 15 kJ/m^2^. MMT and HNT additions suppressed this improvement, demonstrating that a nanofiller can restrict rather than reinforce energy-dissipating deformation when its localization and interfacial effects are unfavorable. In contrast, CNT-containing systems retained impact strengths close to 15 kJ/m^2^, with better thermal resistance. The work carries an important lesson: nanofillers in multiphase polymer blends must be evaluated as modifiers of phase morphology and fracture mechanisms, and are not presumed to impart universal reinforcement.

Zhang et al. introduced size-controllable micro-hydrogels as bacterial analogs for probing filtration within symmetric and non-traditional asymmetric polyethersulfone microporous membranes [38]. Four nearly monodisperse microgels with average diameters of 294, 362, 431, and 517 nm were used to clarify how a graded pore architecture governs particle localization and blocking. In the asymmetric membrane, the 294 and 362 nm microgels were retained near pore throats, whereas the 431 and 517 nm particles showed partial penetration before accumulating within pores or on the surface. For the 362 nm microgel system, the retention was approximately 54% at solid concentrations below 30 mg L^−1^, increasing to 75.3% at 90 mg L^−1^ with cake layer development. Model analysis linked the 362 nm and 431 nm microgels to adsorption and standard blocking, respectively. This controlled surrogate approach reveals structure-dependent fouling and guides membrane selection in practical biopharmaceutical and biological separation.

Doan et al. examined ZnO particles modified with M8 (a blend of chitosan, poly(vinyl alcohol), poly(vinylpyrrolidone), and poly(ethylene glycol)) [39]. Scanning electron microscopy revealed the mean size of unmodified ZnO particles to be 181.8 nm. Antibacterial assays revealed that the responses of M8 and M8/ZnO against *Staphylococcus aureus*, *Salmonella enterica*, and *Pseudomonas aeruginosa* were bacterial-species-dependent. ZnO incorporation did not consistently improve the inhibitory activity of the polymer blend. Notably, M8/ZnO did not achieve 90% inhibition of *P. aeruginosa* under the tested conditions. Hence, the antibacterial performance must be assessed for each formulation and organism, with polymer/particle ratio optimization being an important direction for further work.

Li et al. combined dispersion control with digital light processing to develop conductive photocurable CNT/polymer composites for capacitive sensing and touch interfaces [40]. SMA-amide dispersants were designed to stabilize the CNT dispersions. Dynamic light scattering revealed that SMA-D2000 (dispersant-to-CNT mass ratio of 1:1) had a mean dispersed particle size of 215.4 nm in tetrahydrofuran; this characterized the liquid dispersion rather than the CNT dimensions in the cured composite. Measurable conduction was observed at ~0.05 wt% CNT, consistent with the onset of network formation, and the resistivity reached (4.2 ± 2.8) × 10^3^ Ω·cm at 0.30 wt% CNT. The optimized formulation increased the ultimate stress by 16%, with a 23% reduction in strain relative to the neat resin. Printed touch interfaces demonstrated the practical correlation between formulation, printing conditions, and customizable device geometry.

## 3. Conclusions

The aforementioned seven contributions demonstrate how polymer composition and processing govern the optical, mechanical, electrochemical, filtration, antibacterial, and sensing performances. The functional properties depend not only on constituent selection but also on material organization, dispersion, interfacial interactions, and pore architecture. These effects are evident in the concentration-dependent optical response of PVDF hybrids, keratin–halloysite reinforcement of bio-polyamide, and the porous structures of electrodes and membranes. The printable CNT system further highlights the importance of dispersion control for fabricating functional polymer components. The studies also emphasize the need for balancing competing properties. Nanofillers that enhance one performance metric may compromise toughness in another matrix, while ZnO incorporation did not consistently improve the antibacterial response of the M8 formulation. Thus, application-specific optimization is preferable to assuming that higher filler loading or additional components will universally improve performance. Meaningful comparisons should consider formulation, loading, processing and testing conditions, and experimental uncertainty. Future research should link laboratory-scale performance with practical service requirements, including aging stability, fatigue/impact resistance, electrochemical performance in complete devices, membrane fouling/regeneration, and sensor hysteresis under various environments. Antibacterial systems also require standardized, organism-specific testing together with assessments of compatibility and particle release. Mechanistic characterization and data-driven approaches may reveal the relationships between interfacial evolution and functional performance when supported by well-defined experimental datasets. Scale-up and sustainability should be considered alongside technical performance. Biomass-derived reinforcements and electrodes allow for resource valorization, but their environmental benefits depend on processing energy, solvent use, durability, and end-of-life management. Life-cycle assessments, reproducible manufacturing, and industrial collaboration are essential for transforming multifunctional polymer materials into reliable and scalable technologies.

## Data Availability

The data presented in this study are available on request from the corresponding author.

## References

[1] Wang S., Luo Z., Liang J., Hu J., Jiang N., He J., Li Q. (2022). Polymer Nanocomposite Dielectrics: Understanding the Matrix/Particle Interface. ACS Nano.

[2] Ahn C., Kim S.-M., Jung J.-W., Park J., Kim T., Lee S.E., Jang D., Hong J.-W., Han S.M., Jeon S. (2018). Multifunctional Polymer Nanocomposites Reinforced by 3D Continuous Ceramic Nanofillers. ACS Nano.

[3] Zhao Z., Fang R., Rong Q., Liu M. (2017). Bioinspired Nanocomposite Hydrogels with Highly Ordered Structures. Adv. Mater..

[4] Chen J., Huang X., Sun B., Jiang P. (2019). Highly Thermally Conductive Yet Electrically Insulating Polymer/Boron Nitride Nanosheets Nanocomposite Films for Improved Thermal Management Capability. ACS Nano.

[5] Qin M., Xu Y., Cao R., Feng W., Chen L. (2018). Efficiently Controlling the 3D Thermal Conductivity of a Polymer Nanocomposite via a Hyperelastic Double-Continuous Network of Graphene and Sponge. Adv. Funct. Mater..

[6] Li X., Zhang Z., Zhang X., Cao Y., Yang Y., Wang W., Wang J. (2024). A Polymer Nanocomposite with Strong Full-Spectrum Solar Absorption and Infrared Emission for All-Day Thermal Energy Management and Conversion. Adv. Sci..

[7] Ren J., Huang C., Tan R., Chen J., Huang M., Wang M., Liu W., Li B., Ma Z., Wang L. (2025). All-Biomass Nanocomposite Films via Facile and Sustainable Design Procedure for Thermal Management and Electromagnetic Interference Shielding. Adv. Sci..

[8] Liu J., Ji L., Yu J., Ding S., Luo S., Chu B., Xu J., Sun R., Yu S. (2023). Enhanced Breakdown Strength and Electrostatic Energy Density of Polymer Nanocomposite Films Realized by Heterostructure ZnO–ZnS Nanoparticles. Chem. Eng. J..

[9] Tang S., Guo W., Fu Y. (2021). Advances in Composite Polymer Electrolytes for Lithium Batteries and Beyond. Adv. Energy Mater..

[10] Vijayakumar V., Ghosh M., Asokan K., Sukumaran S.B., Kurungot S., Mindemark J., Brandell D., Winter M., Nair J.R. (2023). 2D Layered Nanomaterials as Fillers in Polymer Composite Electrolytes for Lithium Batteries. Adv. Energy Mater..

[11] Zhu Q., Wang X., Miller J.D. (2019). Advanced Nanoclay-Based Nanocomposite Solid Polymer Electrolyte for Lithium Iron Phosphate Batteries. ACS Appl. Mater. Interfaces.

[12] Wang F., Zhang Z., Shakir I., Yu C., Xu Y. (2022). 2D Polymer Nanosheets for Membrane Separation. Adv. Sci..

[13] Baig M.I., Durmaz E.N., Willott J.D., de Vos W.M. (2020). Sustainable Membrane Production through Polyelectrolyte Complexation Induced Aqueous Phase Separation. Adv. Funct. Mater..

[14] Aashika R.C., Sumi K., Prasath J., Arivanandhan M. (2025). Bifunctional Characteristics of *Vetiveria zizanioides*-Incorporated PVDF Polymer Nanocomposite Membrane for Wastewater Treatment and Energy Storage Applications. Chem. Eng. J..

[15] Chen J.-X., Li J.-W., Jiang Z.-J., Chiu C.-W. (2024). Polymer-Assisted Dispersion of Reduced Graphene Oxide in Electrospun Polyvinylidene Fluoride Nanofibers for Enhanced Piezoelectric Monitoring of Human Body Movement. Chem. Eng. J..

[16] Li J.-W., Huang C.-Y., Zhou B.-H., Hsu M.-F., Chung S.-F., Lee W.-C., Tsai W.-Y., Chiu C.-W. (2022). High Stretchability and Conductive Stability of Flexible Hybrid Electronic Materials for Smart Clothing. Chem. Eng. J. Adv..

[17] Chen S., Wang Y., Fei B., Long H., Wang T., Zhang T., Chen L. (2022). Development of a Flexible and Highly Sensitive Pressure Sensor Based on an Aramid Nanofiber-Reinforced Bacterial Cellulose Nanocomposite Membrane. Chem. Eng. J..

[18] Li J.-W., Huang B.-S., Chang C.-H., Chiu C.-W. (2023). Advanced Electrospun AgNPs/rGO/PEDOT:PSS/TPU Nanofiber Electrodes: Stretchable, Self-Healing, and Perspiration-Resistant Wearable Devices for Enhanced ECG and EMG Monitoring. Adv. Compos. Hybrid Mater..

[19] Yoo J.-Y., Seo M.-H., Lee J.-S., Choi K.-W., Jo M.-S., Yoon J.-B. (2018). Industrial Grade, Bending-Insensitive, Transparent Nanoforce Touch Sensor via Enhanced Percolation Effect in a Hierarchical Nanocomposite Film. Adv. Funct. Mater..

[20] Suo J., Liu Y., Wu C., Chen M., Huang Q., Liu Y., Yao K., Chen Y., Pan Q., Chang X. (2022). Wide-Bandwidth Nanocomposite-Sensor Integrated Smart Mask for Tracking Multiphase Respiratory Activities. Adv. Sci..

[21] Fang Y., Xu J., Xiao X., Zou Y., Zhao X., Zhou Y., Chen J. (2022). A Deep-Learning-Assisted On-Mask Sensor Network for Adaptive Respiratory Monitoring. Adv. Mater..

[22] Wang M., Wang T., Luo Y., He K., Pan L., Li Z., Cui Z., Liu Z., Tu J., Chen X. (2021). Fusing Stretchable Sensing Technology with Machine Learning for Human-Machine Interfaces. Adv. Funct. Mater..

[23] Chen K., Li M., Yang Z., Ye Z., Zhang D., Zhao B., Xia Z., Wang Q., Kong X., Shang Y. (2024). Ultra-Large Stress and Strain Polymer Nanocomposite Actuators Incorporating a Mutually Interpenetrated, Collective-Deformation Carbon Nanotube Network. Adv. Mater..

[24] Li J.-W., Chen H.-F., Liu Y.-Z., Wang J.-H., Lu M.-C., Chiu C.-W. (2024). Photocurable 3D-Printed AgNPs/Graphene/Polymer Nanocomposites with High Flexibility and Stretchability for ECG and EMG Smart Clothing. Chem. Eng. J..

[25] Palaganas J.O., Palaganas N.B., Ramos L.J.I., David C.P.C. (2019). 3D Printing of Covalent Functionalized Graphene Oxide Nanocomposite via Stereolithography. ACS Appl. Mater. Interfaces.

[26] Stouten J., Schnelting G.H.M., Hul J., Sijstermans N., Janssen K., Darikwa T., Ye C., Loos K., Voet V.S.D., Bernaerts K.V. (2023). Biobased Photopolymer Resin for 3D Printing Containing Dynamic Imine Bonds for Fast Reprocessability. ACS Appl. Mater. Interfaces.

[27] Ahmed A., Adak B., Bansala T., Mukhopadhyay S. (2020). Green Solvent Processed Cellulose/Graphene Oxide Nanocomposite Films with Superior Mechanical, Thermal, and Ultraviolet Shielding Properties. ACS Appl. Mater. Interfaces.

[28] Ge W., De Silva R., Fan Y., Sisson S.A., Stenzel M.H. (2025). Machine Learning in Polymer Research. Adv. Mater..

[29] Kim J.Y., Koh E.H., Yang J.-Y., Mun C., Lee S., Lee H., Kim J., Park S.-G., Kang M., Kim D.-H. (2024). 3D Plasmonic Gold Nanopocket Structure for SERS Machine Learning-Based Microplastic Detection. Adv. Funct. Mater..

[30] Chen Y.-F., Lee Y.-C., Lee J.C.-M., Li J.-W., Chiu C.-W. (2024). Coaxial Electrospinning of Au@Silicate/Poly(vinyl alcohol) Core/Shell Composite Nanofibers with Non-covalently Immobilized Gold Nanoparticles for Preparing Flexible, Freestanding, and Highly Sensitive SERS Substrates Amenable to Large-Scale Fabrication. Adv. Compos. Hybrid Mater..

[31] Lin L.L., Alvarez-Puebla R., Liz-Marzán L.M., Trau M., Wang J., Fabris L., Wang X., Liu G., Xu S., Han X.X. (2025). Surface-Enhanced Raman Spectroscopy for Biomedical Applications: Recent Advances and Future Challenges. ACS Appl. Mater. Interfaces.

[32] Lu M.-C., Lee C.-J., Yao C.-Y., Wu B.-Y., Chiu C.-W. (2026). Dynamic Surface-Enhanced Raman Scattering Platform with Multiscale Nanostructural Engineering and Programmable Surface Energy for Artificial Intelligence-Assisted Discrimination of *Helicobacter pylori*. Adv. Oncol..

[33] Li J.-W., Cheng C.-C., Chiu C.-W. (2024). Advances in Multifunctional Polymer-Based Nanocomposites. Polymers.

[34] Taha T.A.M., Alanazi S.S., El-Nasser K.S., Alshammari A.H., Ismael A. (2024). Structure–Property Relationships in PVDF/SrTiO_3_/CNT Nanocomposites for Optoelectronic and Solar Cell Applications. Polymers.

[35] Teodorescu G.M., Vuluga Z., Ioniță A., Nicolae C.A., Ghiurea M., Gabor A.R., Rădițoiu V., Raduly M., Brezeștean I.A., Marconi D. (2024). Morphological, Thermal, and Mechanical Properties of Nanocomposites Based on Bio-Polyamide and Feather Keratin–Halloysite Nanohybrid. Polymers.

[36] Tran N., Choi H.W., Tran Q.N. (2024). A Review of Green Aerogel- and Xerogel-Based Electrodes for Supercapacitors. Polymers.

[37] Markowski M., Piasecki A., Andrzejewski J. (2025). The Application of Montmorillonite (MMT), Halloysite (HNT), and Carbon Nanotubes (CNT) in Toughened Polyethylene Terephthalate Glycol/Polycarbonate (PETG/PC) Blends: The Critical View on the Use of Nanosized Fillers as Phase Structure Modifiers. Polymers.

[38] Zhang H., Zhu T., Zheng Y., Liu W., Zhang T., Mao Y., Wang J., Zhu L., Xu C., Wang J. (2025). Detection of Filtration Characteristics of Nontraditional Asymmetric Microporous Membranes Using Size-Controllable Micro-Hydrogel. Polymers.

[39] Doan L., Tran K., Huynh K.G., Nguyen T.M.D., Tang L.V.H. (2025). Surface Modifications of Zinc Oxide Particles with Chitosan, Polyethylene Glycol, Polyvinyl Alcohol, and Polyvinylpyrrolidone as Antibacterial Agents. Polymers.

[40] Li J.-W., Chen H.-F., Huang P.-H., Kuo C.-F.J., Cheng C.-C., Chiu C.-W. (2023). Photocurable Carbon Nanotube/Polymer Nanocomposite for the 3D Printing of Flexible Capacitive Pressure Sensors. Polymers.

